# Load Dependency of Postural Control - Kinematic and Neuromuscular Changes in Response to over and under Load Conditions

**DOI:** 10.1371/journal.pone.0128400

**Published:** 2015-06-08

**Authors:** Ramona Ritzmann, Kathrin Freyler, Elmar Weltin, Anne Krause, Albert Gollhofer

**Affiliations:** Institute of Sport and Sport Science, University of Freiburg, Freiburg, Germany; West Virginia University, UNITED STATES

## Abstract

**Introduction:**

Load variation is associated with changes in joint torque and compensatory reflex activation and thus, has a considerable impact on balance control. Previous studies dealing with over (OL) and under loading (UL) used water buoyancy or additional weight with the side effects of increased friction and inertia, resulting in substantially modified test paradigms. The purpose of this study was to identify gravity-induced load dependency of postural control in comparable experimental conditions and to determine the underlying neuromuscular mechanisms.

**Methods:**

Balance performance was recorded under normal loading (NL, 1g), UL (0.16g; 0.38g) and OL (1.8g) in monopedal stance. Center of pressure (*COP*) displacement and frequency distribution (low 0.15-0.5Hz (*LF*), medium 0.5-2Hz (*MF*), high 2-6Hz (*HF*)) as well as ankle, knee and hip joint kinematics were assessed. Soleus spinal excitability was determined by H/M-recruitment curves (*H/M-ratios*).

**Results:**

Compared to NL, OL caused an increase in ankle joint excursion, *COP HF* domain and *H/M-ratio*. Concomitantly, hip joint excursion and *COP LF* decreased. Compared to NL, UL caused modulations in the opposite direction: UL decreased ankle joint excursions, *COP HF* and *H/M-ratio*. Collaterally, hip joint excursion and *COP LF* increased. *COP* was augmented both in UL and in OL compared to NL.

**Conclusion:**

Subjects achieved postural stability in OL and UL with greater difficulty compared to NL. Reduced postural control was accompanied by modified balance strategies and compensatory reflex activation. With increasing load, a shift from hip to ankle strategy was observed. Accompanying, *COP* frequency distribution shifted from *LF* to *HF* and spinal excitability was enhanced. It is suggested that in OL, augmented ankle joint torques are compensated by quick reflex-induced postural reactions in distal muscles. Contrarily, UL is associated with diminished joint torques and thus, postural equilibrium may be controlled by the proximal segments to adjust the center of gravity above the base of support.

## Introduction

The upright stance is organized by the central nervous system (CNS) using sensory information from the visual, vestibular and proprioceptive afferents [1–4]. Studies dealing with postural control demonstrated that the human body acts as an inverted pendulum [5] using muscle force to provide an appropriate torque (1) counteracting the gravitational force in the vertical plane and (2) compensating for an immediate deterioration of balance control caused by center of gravity (COG) shifts in the horizontal plane. Changes in posture subsequently require an adequate level of muscle force acting against the external forces to restore a given point of equilibrium.

From literature it is well known that postural control is load-dependent [5]. Previous studies dealing with over (OL) and under loading (UL) used water buoyancy [6,7], partial weight-bearing [8–11], free fall conditions [12,13], hypergravity [12] or extra weight [8,6]. In these studies it has been demonstrated that load variation has a considerable impact on balance control and is associated with changes in joint torque [5,7], somatosensory feedback [14–16] and neuromuscular activity [8,17,6,18]. Not least, space research on astronauts has demonstrated that load-dependent adaptations are accompanied by distinct changes in motor control, neuromuscular activity and compensatory reflex activation [15] and it is questioned whether applications for counteractive gravity tactics could compensate for these modulations [19,20]. However, despite the substantial amount of load-related articles, the underlying neuromuscular mechanisms and functional consequences for balance control are poorly understood.

Previous studies investigating the effect of load on ***biomechanical aspects of postural control*** focused on OL paradigms and reported an overall increase in center of pressure (COP) displacement [21–25], COP velocity [25] and COP mean frequency distribution [26] in response to additional load attached to various body segments. Authors suggest that these modulations are caused by load-induced shifts in balance strategy [21,26]. Importantly, the COP time and frequency characteristics [21,26] as well as the balance strategy [21] were substantially influenced by the distance of the load from the COG in the horizontal plane and height in the vertical plane [27,21,28]. It is concluded that due to the high sensitivity of postural control in terms of loading modalities, the application point of loading within the test setting is of considerable importance for the outcomes of the studies. In UL, no studies have been executed yet.

Investigations assessing the effect of load on the ***neuromuscular system*** focused on the reflex arc that controls the operation of immediate reflexive muscle contractions, which are considered to be of major relevance for a quick readjustment of the body segments to restore equilibrium in a balance task [6]. However, although extensive research was executed on the load dependency of reflexes, the documented adaptations following OL and UL are inconsistent in literature. On the one hand, some authors reported UL-induced reflex facilitation [12,7,13], which was argued to be associated with a decline in presynaptic inhibition due to altered somatosensory afferent input [7]. Contrarily, other authors could not confirm these results and demonstrated an inhibition of the soleus H-reflex [6,10,18] and stretch reflex response [18] due to UL induced by water buoyancy and partial weight-bearing. Finally, Phadke et al. [11] and Ali and Sabbahi [8] did not observe any differences in response to UL. Likewise, studies conducted in OL conditions revealed opposing outcomes ranging from a reflex facilitation [12], to a reflex inhibition [7], to no effect at all [8,6]. To explain the contradictory results, it is emphasized that differences in methodologies and test settings among the various studies conducted under water, with partial weight-bearing or additional load may have contributed to the opposing findings. Side effects such as increased friction [6], superficial stabilization [17], hydrostatic pressure [18,29] and inertia [5] may have led to considerably modified test paradigms [30] in the analysis from UL to OL. As a consequence, the effect of load variation on neuromuscular control is still poorly understood.

To minimize negative side effects occurring beyond load variation, the most reliable test conditions can be achieved in space-like environments by a gradual change of the gravitational force. As shown by modeling studies [5], an approach that provides reliable test settings for OL and UL could be provided by modulated gravitational accelerations while keeping other side factors (i.e. inertia or friction) constant. As the body mass remains constant, the only variable that alters the force acting on the human body and thus defines the magnitude of load is the gravitational acceleration (F = m*a). As a further advantage, methodological difficulties regarding the application points of loading are moot, as OL and UL are equally distributed among the body segments. Parabolic flights offer such reliable research conditions, in which the gravitational forces are progressively increased within a range from UL in Lunar (0.16g), via Martian (0.38g) to earth (1g) to OL in hypergravity (1.8g) conditions. Furthermore, the effect of artificial loading can be evaluated in terms of countermeasures testing the suitability of exercise regimens to counteract degenerations caused by weightlessness [19,15,20].

Therefore, the objective of this study was to elucidate the gravity-induced load dependency in balance control. For that purpose, we aimed to (1) identify modulations in balance control and strategy based on a neuromuscular and kinematic assessment in UL and OL and to (2) gather knowledge about the neuro-mechanical coupling in terms of loading. Additionally, we (3) explored the application of artificial loading under zero gravity conditions to evaluate possible compensatory functions for the lack of gravitational force as a countermeasure application in space.

## Materials and Methods

### Subjects

Eight subjects (2 female, 6 male, height 177±7cm, body mass 69±7kg, age 31±4years) participated in this study. All participants gave written informed consent to the experimental procedure, which was in accordance with the latest revision of the Declaration of Helsinki and approved by the French authorities responsible for the protection of subjects participating in biomedical research (DEMEB of the AFSSAPS) as well as the ethics committee of the University of Freiburg. The participants were healthy with no previous neurological irregularities or injuries of the lower extremities. Inclusion criteria were: no motion sickness and a reliable H-reflex. Six subjects were experienced flyers; two were first flyers.

### Parabolic flight

**Fig 1:**
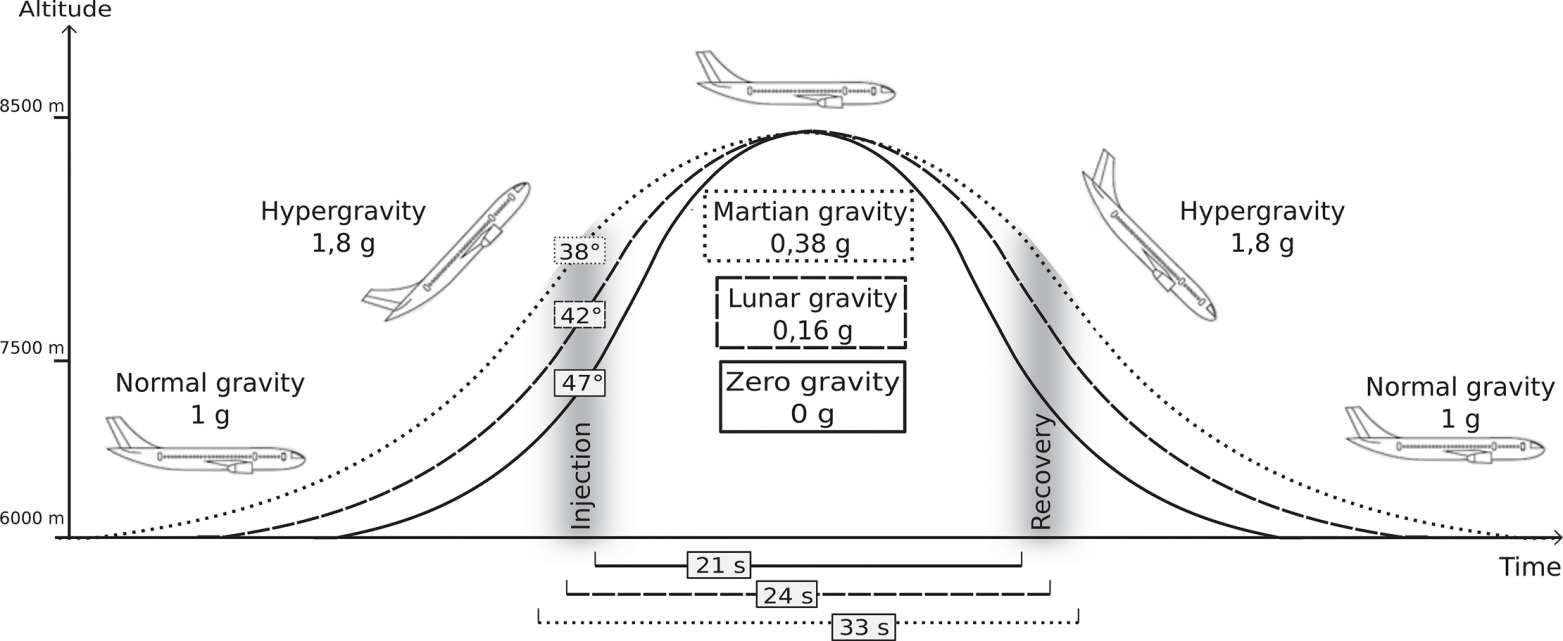
Parabolic flight maneuver and the corresponding acceleration levels according to the aircraft’s angle of attack: the flight level (normal gravity) is interrupted by a steep climb flight inducing 20s of hypergravity, followed by 33s of Martian, 24s Lunar or 21s zero gravity and another hypergravity phase before returning to a normal gravity flight level again. "Injection" marks the transition from hypergravity to Lunar /Martian/zero gravity (duration ca. 1s) and "recovery" marks the transition from Lunar /Martian/zero gravity to hypergravity (duration 1s) at the end of the free fall. The different gravitational conditions were used to assess postural control in various unloading and overloading conditions.

The experiment was conducted aboard the Airbus A300 ZERO-G (Novespace, Bordeaux, France) during the first and second CNES/ESA/DLR Joint European Partial-g Parabolic Flight Campaign (JEPPF-1 and 2). The campaign comprised four flight days, each flight lasted three hours and comprised 13 Martian, 12 Lunar and 6 zero gravity parabolas. Data from a total of 104 parabolas were analyzed (at least 3 Lunar, 3 Martian and 3 zero gravity parabolas per subject). The course of one parabola is illustrated in Fig 1: each of the parabolas started from a horizontal flight and typically included two hypergravity (HG, 1.8g) periods of 20s, separated by a 21s zero gravity (ZG, 0g), a 24s Lunar gravity (LG, 0.16g) or a 33s Martian gravity (MG, 0.38g) period, respectively. The flight protocol was identical for each subject and all flight days and the order of the parabolas was not randomized. The normal gravity (NG) phase between two parabolas lasted between 1 and 8 min and was used as a pause for auxiliary procedures and to switch subjects.

### Experimental design

**Fig 2:**
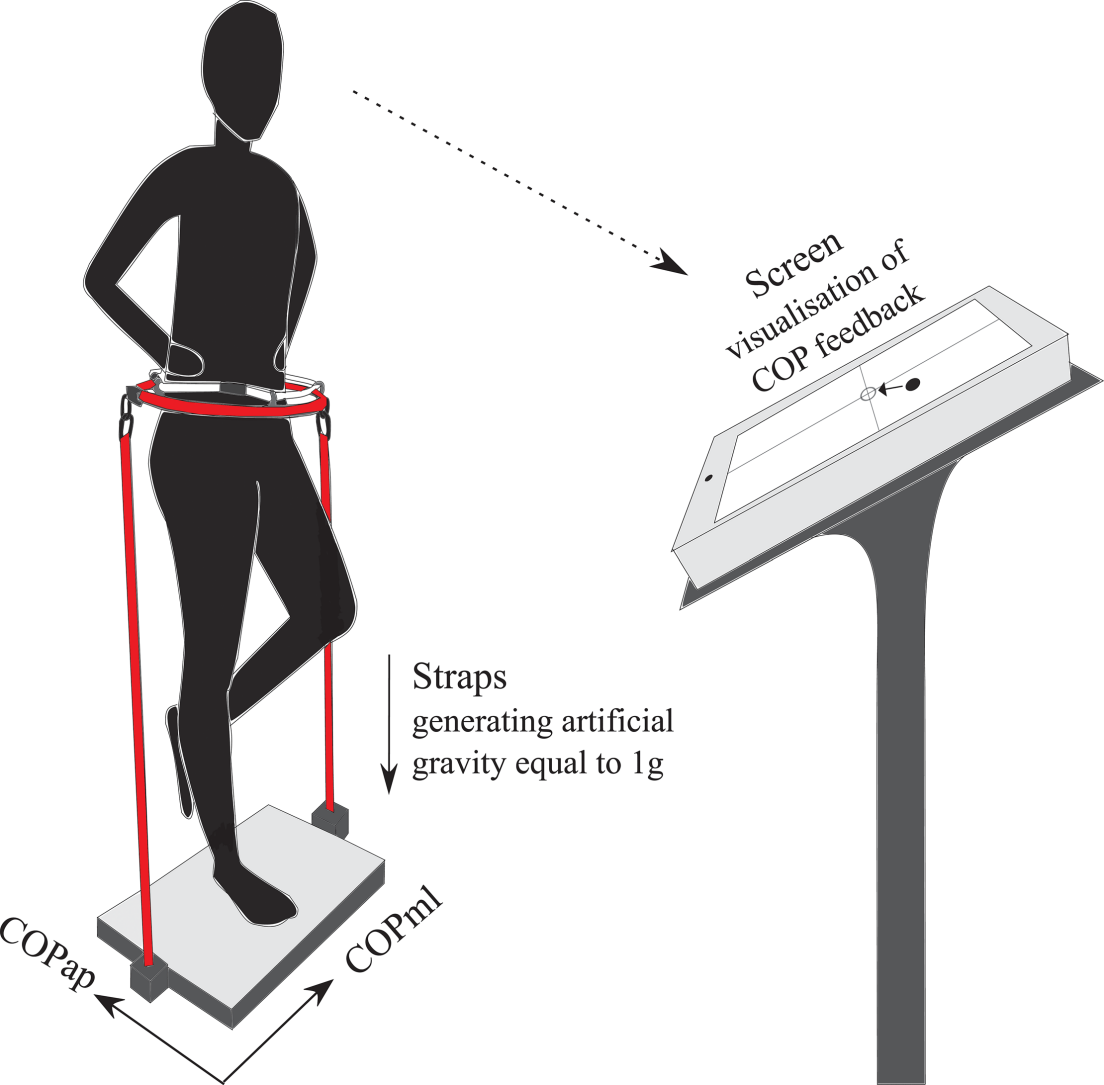
Experimental setting: Artificial loading (loading equal to 1g) was achieved by means of a harness affixed around the subject’s hip and attached to the floor via springs close to subject’s lateral sides of the right foot. The oil-ring provides freedom of movement to avoid stabilizing effects. In all test conditions, subjects received a COP feedback visualized on a screen in front of the experimental setup.

A single-group repeated-measures study design was used to assess load-dependent changes in postural and neuromuscular control in five different conditions: during normal loading (NL in NG conditions; 1g), simulated NL (S_NL; superficially loaded (S_NG) to NL in ZG, Fig 2), in UL under LG and MG conditions and in OL under HG while maintaining postural equilibrium in monopedal stance. In all load conditions, the subjects performed a one-legged balance task on their right leg, barefoot, knees extended, eyes open and hands akimbo [31]. This paradigm was chosen as it provides a reliable training-relevant but challenging test setting without the interference of asymmetries or unequal weight distribution as it can occur in bipedal stance [32,33]. During the measurements, subjects received visual COP feedback and were instructed to hold their COP (displayed on a screen in front of the subject, distance 2m) centered and as still as possible.

All subjects were experienced in balance training, were introduced to the test procedures before and trained the balance task with COP feedback the day prior to measurements to assure repeatability and reproducibility.

On ground before each flight day, two participants were prepared for the experiment and were given an injection of 0.2–0.7ml of Scopolamine one hour before take-off to prevent motion sickness [34]. To control for confounding side effects of Scopolamine [35], the dose was limited to the minimum and injection was given as early as possible, so that none of the subjects reported drowsiness or fatigue during the measurements [36]. Isometric maximal voluntary contractions (MVC) were performed for all recorded muscles according to Roelants et al. [37].

#### Postural sway

Postural sway was quantified by means of the COP displacement on a sensor mat (Pedar Posturo System, Novel, Germany). The COP displacement was recorded with a frequency of 100Hz and the overall COP displacement in medial–lateral (COP_ml_) and anterior–posterior (COP_ap_) direction as well as frequency characteristics were assessed and averaged over the trials for each subject and each of the five conditions [38]. In view of literature, spectral energy is distributed in three frequency bands depending on the type of somatic regulation in the frequency analysis of equilibrium: low frequencies (0.15–0.5Hz) are related to the action of the sensory systems (visual and vestibular), medium frequencies (0.5–2Hz) correspond to the regulation of the cerebellum, and high frequencies (2-6Hz) reflect proprioceptive regulation induced by reflexes [39,38,40].

#### Kinematics

Ankle (dorsiflexion and plantarflexion), knee (flexion and extension) and hip (extension and flexion) joint kinematics in the sagittal plane were recorded by electrogoniometers (Biometrics, Gwent, UK). The ankle goniometer was fixed at the lateral aspect of the right ankle with its ends attached parallel to the major axis of the foot in line with the fifth metatarsal and the major axis of the leg in line with the fibula. The knee goniometer was placed over the lateral epicondyle of the femur with one endplate attached to the shank and aligned to the lateral malleolus of the fibula and the other to the thigh aligned to the greater trochanter. For the hip, one endplate was fixed to the lateral pelvis midline and the other to the greater trochanter of the femur. The knee and hip flexion angle was set to zero at 0° during normal upright stance, joint flexion was reflected by an angle greater than 0°. An angle of 90° between the fifth metatarsal and the fibula was defined as a 90° ankle angle; a plantar flexion was reflected by an angle greater than 90°. All signals were filtered (band-pass filter 10Hz to 1kHz) and recorded with a sampling frequency of 1kHz.

#### Electromyography (EMG)

Bipolar Ag/AgCl surface electrodes (Ambu Blue Sensor P, Ballerup, Denmark, diameter 9 mm, center-to-center distance 34 mm) were placed over the soleus (SOL), medial gastrocnemius (GM), tibialis anterior (TA), rectus femoris (RF), biceps femoris (BF), vastus lateralis (VL) and gluteus maximus (GL) muscles of the right leg. The longitudinal axes of the electrodes were in line with the presumed direction of the underlying muscle fibers. The reference electrode was placed on the patella. Interelectrode resistance was kept below 2 kΩ by means of shaving, light abrasion and degreasing of the skin with a disinfectant. The EMG signals were transmitted via shielded cables to the amplifier (band-pass filter 10Hz to 1kHz, 200x amplified) and recorded with 1kHz (A/D-conversion via a National Instruments PCI-6229 DAQ-card, 16bit resolution).

#### Peripheral nerve stimulation

Peripheral nerve stimulation (PNS) was executed in all subjects during each of the measurements in each of the five conditions on the standing leg. H-reflexes were elicited by PNS to assess load-induced modulations in spinal excitability of the SOL motoneuron pool. An electrical stimulator (Digitimer DS7, Digitimer, Welwyn Garden City, UK) was used to generate rectangular pulses of 1ms duration. The cathode (2cm in diameter) was placed in the popliteal fossa and moved until the best position was found for eliciting an H-reflex in the SOL. The anode (10*5cm dispersal pad) was fixed below the patella on the anterior aspect of the knee. H-reflexes were elicited by electrically stimulating the posterior tibial nerve with an interstimulus interval of four seconds. The stimulation current was successively augmented, ranging from subthreshold stimulation intensities to intensities sufficient to elicit H-reflexes to supramaximal intensities for the maximal M-wave [31]. At least 30 stimulations were necessary to obtain one H/M-recruitment curve during each of the load levels.

### Data processing

For each of the five gravity levels, COP displacement and frequency characteristics were established: COP_ml_ and COP_ap_ were assessed, averaged over the trials for one subject and time normalized. To assess the involvement of short or long neuronal loops in equilibrium regulation, the COP frequency domain was calculated according to Cabeza-Ruiz et al. [38]. Thus, Fast Fourier Transforms were applied to the COP signal and the spectrum was calculated between 0.15 and 6Hz (resolution = 0.025 Hz). The total spectral energy (TSE) was calculated and categorized among three frequencies as follows: low frequencies (LF within a range of 0.15–0.5Hz), medium frequencies (MF, 0.5-2Hz) and high frequencies (HF, 2-6Hz). The values of each of these three frequency bands were expressed as a percentage of TSE.

Kinematics were expressed as mean ankle, knee and hip angles [°] and mean joint excursions (change in joint angle per second averaged over the recording time) [°/s], respectively.

For each of the recorded muscles in each load condition, EMG signals were rectified, integrated and time normalized (iEMG [mVs]). For SOL, the pre-activation 100ms prior to PNS was determined and expressed as iEMG [mVs]) to control for possible changes in the background EMG in regard to the validity of the H-reflex recordings. In addition, to assess the simultaneous activation of antagonistic muscles encompassing the ankle, knee and hip joint, the co-contraction index (CCI) was calculated for TA_SOL, BF_VL and RF_GL with the rectified and normalized EMG by means of the following equation: CCI_i_ = Σ(lower EMG_i_/higher EMG_i_)x(lower EMG_i_+higherEMG_i_) for each sample point, CCI = Σ CCI_i_ [41].

Furthermore, for each of the five load levels peak-to-peak amplitudes of the H-reflexes and M-waves were calculated and the maximal H-reflex (H_max_) was expressed as relative to the maximal M-wave (M_max_), defined as H_max_/M_max_-ratios.

### Statistics

The mean values for each load condition and participant were used for statistical analysis. Statistical differences regarding the different gravity conditions (LG, MG, NG and HG) were determined by non-parametric analysis (Friedman ANOVA). In case of significant findings, pairwise comparison between NG and LG (MG and HG, respectively) were calculated (Wilcoxon signed-rank test); Bonferroni was used to correct for multiple testing. The level of significance was set to P = 0.05, and statistically significant differences were marked with a (*) symbol.

In addition, equivalence statistics were used to determine if the S_NG values were statistically equal to NG values. For this purpose, the 90% confidence interval (CI) was calculated for the differences between the two load conditions. If the CI lay within the acceptable boundaries (which were determined based on the variance within the NG data set [42]) the differences were statistically equal and the respective parameter was marked with a (≈). Statistical analyses were conducted using the SPSS 16.0 software (SPSS Inc., Chicago, Illinois, USA).

A post hoc power analysis was performed retrospectively to assess the statistical power with respect to α = 0.05, n = 8 and effect size = 0.7 with a resulting power (1-β) = 0.8.

Group data are presented as mean±standard deviation (M±SD).

## Results

### Postural sway

**Fig 3:**
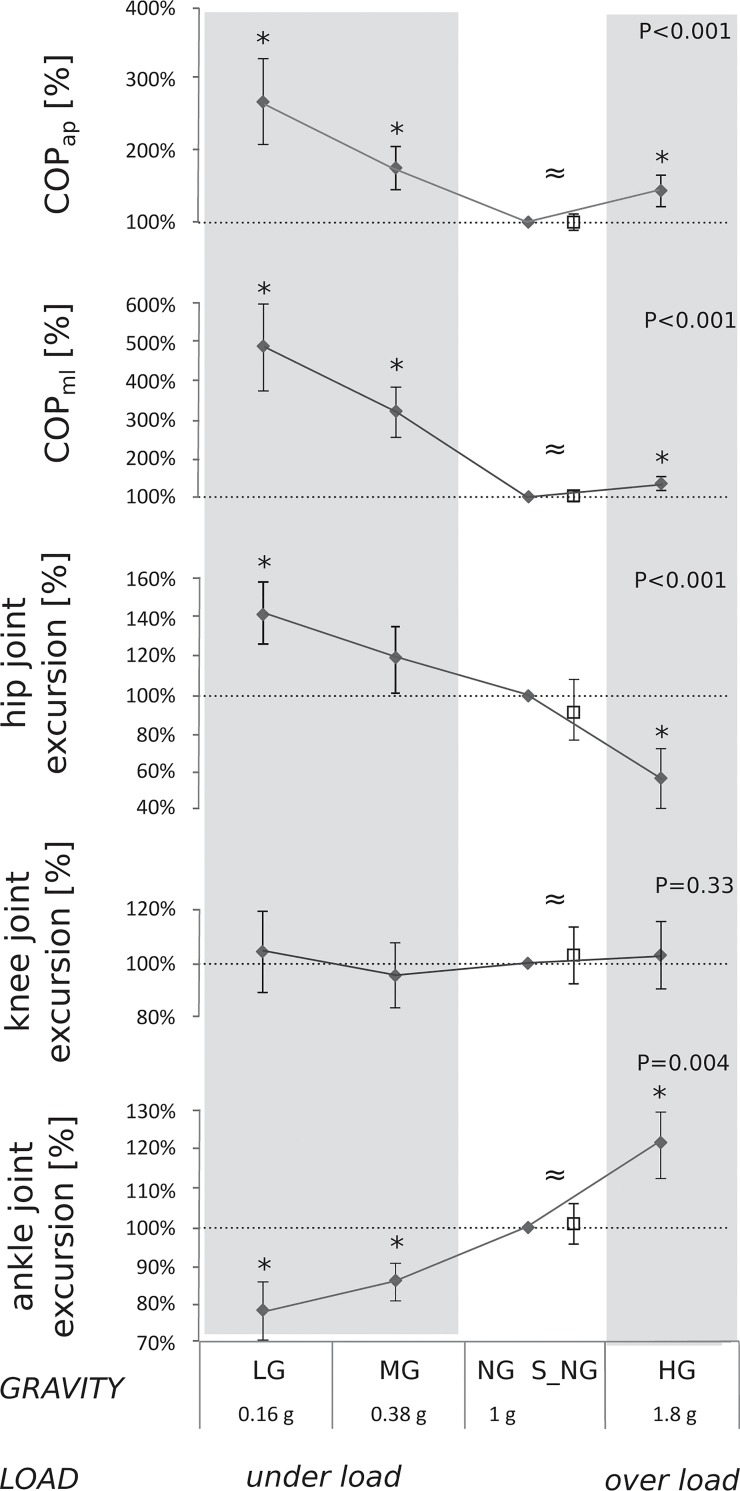
Grand mean of the kinematic parameters in under and over loading collected in four gravity levels (lunar (LG, grey background), Martian (MG, grey), normal gravity (NG, normal loading, white) and hypergravity (HG, grey)) as well as during simulated normal gravity (S_NG, white): Total COP displacement in anterior-posterior and medio-lateral direction (COP_ml_ and COP_ap_) and mean ankle, knee and hip joint excursions. Data are normalized to the respective NG value. Loading has a statistically significant influence on all parameters (P<0.05 denoting a significant ANOVA result, * symbol denotes a statistically significant difference compared to the 1g condition) despite the mean knee joint excursion. Statistical equivalence ≈ between NG and S_NG conditions values could be observed for all parameters.

**Fig 4:**
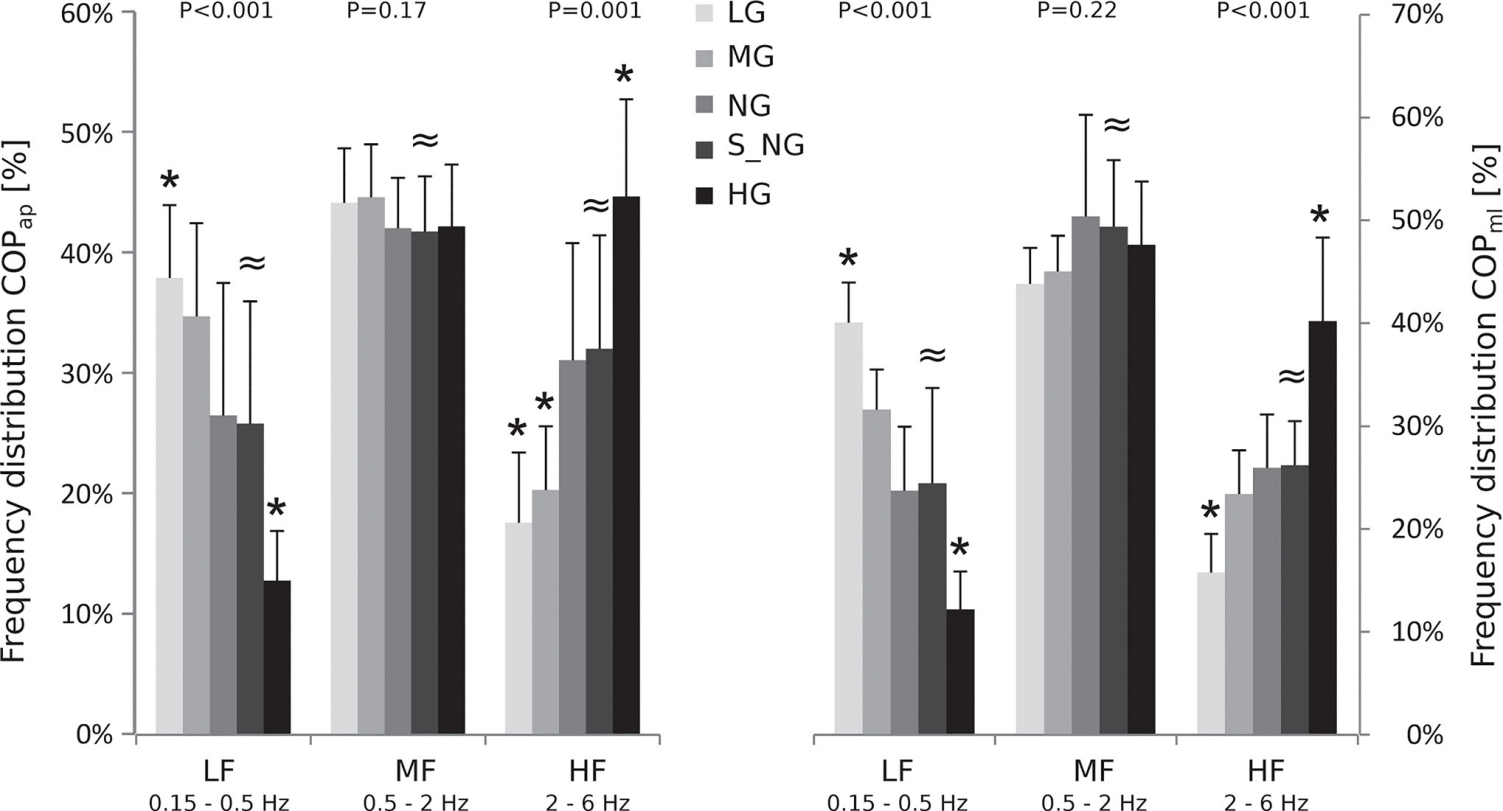
Grand mean of the frequency domains for under and over loading in the four gravity levels (lunar (LG), Martian (MG), normal gravity (NG) and hypergravity (HG)) as well as for the simulated normal loading condition (S_NG): Low frequencies (LF) represent the amount of spectral energy of the frequency spectrum accumulated between 0.15 and 0.5Hz, medium frequencies (MF) the amount accumulated between 0.5 and 2Hz and high frequencies (HF) between 2 and 6Hz. Gravity has a statistically significant influence on HF and LF (P<0.05, * symbol denotes a statistically significant difference compared to the NG condition). Statistical equivalence ≈ between NG and simulated normal loading (S_NG) conditions values could be observed for LF, MF and HF.

Load-dependent changes in COP characteristics are illustrated in Figs 3 and 4. Significant differences for both COP_ap_ and COP_ml_ were observed as a result of load variation: for the load conditions below and above NL, COP_ap_ and COP_ml_ were distinctly increased compared to NL (Fig 3, Table 1). COP_ap_ and COP_ml_ in S_NL compared to the NL were statistically equal.

**Table 1:** Averaged values of all participants for COP_ap_ and COP_ml_ and the COP frequency distribution (LF 0.15–0.5Hz, MF 0.5-2Hz and HF 2-6Hz expressed as a percentage of TSE) for the gravity conditions below (Lunar (LG) and Martian (MG)) and above (hypergravity (HG)) normal gravity (NG).

Change [%]	LG *(0.16g)*	MG *(0.38g)*	NG (1g)	*S_NG*	HG *(1.8g)*	P	Χ^2^
COP ap	2.94±0.90*	1.92±0.29*	1	*1*.*01*±*0.11≈*	1.42±0.21*	<0.001	24.00
LF ap	0.40±0.06*	0.32±0.07	0.27±0.11	*0.26±0.10≈*	0.12±0.04*	<0.001	21.00
MF ap	0.44±0.05	0.47±0.05	0.42±0.04	*0.42±0.05≈*	0.42±0.05	= 0.17	4.97
HF ap	0.18±0.06*	0.18±0.04*	0.31±0.10	*0.32±0.09≈*	0.49±0.06*	= 0.001	16.90
COP ml	4.36±1.20*	2.96±0.75*	1	*1.02±0.09 ≈*	1.27±0.14*	<0.001	22.95
LF ml	0.40±0.04*	0.32±0.04	0.24±0.06	*0.24±0.09 ≈*	0.12±0.04*	<0.001	20.85
MF ml	0.43±0.06	0.44±0.04	0.50±0.10	*0*.*49±0*.*06*	0.48±0.06	= 0.22	3.86
HF ml	0.18±0.06*	0.25±0.04	0.26±0.05	*0.26±0.04 ≈*	0.40±0.08*	<0.001	20.70

COP data are normalized to NG.

* indicates significant differences (p<0.05) compared to NG.

≈ indicates statistical equivalence for simulated normal gravity (S_NG) compared to NG.

P and Χ^2^ values in the right columns display Friedman ANOVA results.

Regarding the COP frequency domain, the MFs remained unchanged in response to the different load levels, whereas significant modulations could be observed for the LFs and HFs: a gradually reduced load from OL to UL led to an increase of the LFs in the TSE and vice versa; a gradually increased load led to an increase of the HFs (Table 1, Fig 4). The comparison of S_NL to NL revealed statistical equivalence for the LFs and HFs (Table 1, Fig 4).

### Kinematics

Mean ankle, knee and hip joint angles showed no differences in response to load variation (Table 2). Mean ankle joint excursions progressively decreased from OL to UL, whereas mean hip joint excursions gradually increased. Mean knee joint excursions remained unchanged (Fig 3).

**Table 2:** Averaged values of all participants for the mean ankle, knee and hip angles (top) as well as the corresponding joint excursions (bottom) for the gravity conditions below (Lunar (LG) and Martian (MG)) and above (hypergravity (HG)) normal gravity (NG).

	LG *(0.16g)*	MG *(0.38g)*	*NG (1g)*	*S_NG*	HG *(1.8g)*	P	Χ^2^
Hip angle [°]	2±1	1±2	*1±3*	*3±2*	0±3	= 0.78	3.45
Knee angle [°]	5±2	4±2	*5±3*	*5±5*	6±3	= 0.86	7.75
Ankle angle [°]	93±2	95±3	*95±5*	*95±4*	91±7	= 0.49	2.95
Hip joint excursion [°/s]	8.5±1.2*	5.9±1.5	3.2±0.6	*2*.*9±0*.*8*	1.2±0.5*	<0.001	18.60
Knee joint excursion [°/s]	2.0±0.4	1.4±0.6	*1*.*6±0*.*8*	*1.9±0.5 ≈*	2.0±0.7	= 0.33	3.45
Ankle joint excursion [°/s]	1.3±0.4*	4.2±1.0*	*6*.*1±1*.*1*	*6.3±1.2 ≈*	9.5±1.9*	= 0.004	13.11

* indicates significant differences (p<0.05) compared to NG.

≈ indicates statistical equivalence for simulated normal gravity (S_NG) compared to NG.

P and Χ^2^ values in the right columns display Friedman ANOVA results.

**Fig 5:**
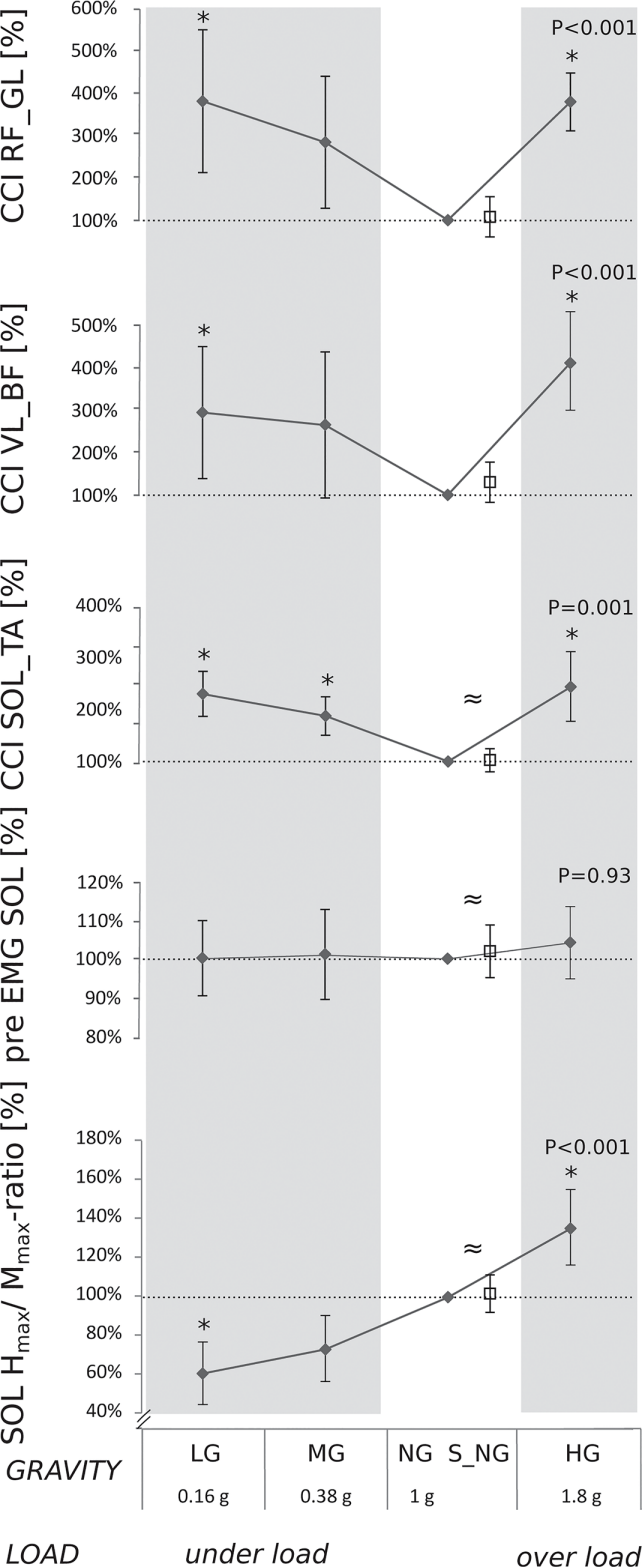
Grand mean of the parameters associated with neuromuscular changes in under and over loading collected in four gravity levels (lunar (LG, grey background), Martian (MG, grey), normal gravity (NG, white) and hypergravity (HG, grey)) as well as during simulated normal gravity (S_NG, white): Co-contraction index (CCI) for the antagonistic muscle groups SOL_TA, BF_VL and RF_GL, H_max_/M_max_-ratios and the iEMG 100ms prior to the H-reflex stimulation for SOL. Data are normalized to the respective NG value. For NG, both the absolute values and the relative values are displayed. Load variation has a statistically significant influence on all parameters (P<0.05, * symbol denotes a statistically significant difference compared to the 1g condition) despite the pre-activation of SOL. Statistical equivalence ≈ between NG and S_NG conditions values could be observed for all parameters despite for CCI BF_VL.

*CCI*: Significant differences in CCI were observed for the antagonistic muscle groups TA_SOL, BF_VL and RF_GL as a result of load variation: for UL and OL conditions the CCIs were distinctly increased compared to NL for all antagonistic muscle groups (Fig 5, Table 3). For TA_SOL, the CCI in S_NL compared to the NL was statistically equal.

**Table 3:** Changes in H_max_/M_max_- ratio, SOL pre-activation and CCI for the antagonistic muscle groups RF_GL, BF_VL and SOL_TA in response to the gravity variation (Lunar (LG), Martian (MG), normal (NG), simulated normal (S_NG) and hypergravity (HG)).

Change [%]	LG *(0.16g)*	MG *(0.38g)*	NG *(1g)*	*S_NG*	HG *(1.8g)*	P	Χ^2^
SOL H_max_/M_max_-ratio	0.61±0.28*	0.73±0.23	1	*1.03±0.10≈*	1.35±0.34*	<0.001	18.60
M_max_	0.97±0.37	0.99±0.45	1	*0*.*98±0*.*37*	0.99±0.40	= 0.27	3.90
pre EMG SOL	1.00±0.24	1.05±0.29	1	*1*.*04±0*.*16*	1.11±0.51	= 0.93	4.50
CCI RF_GL	3.29±1.37*	2.50±1.27	1	*1*.*09±0*.*39*	3.28±0.56*	<0.001	19.95
CCI BF_VL	2.03±0.43*	1.95±0.57	1	*1*.*05±0*.*29*	3.23±0.73*	<0.001	22.20
CCI SOL_TA	2.30±0.43*	1.88±0.37*	1	*1.04±0.22≈*	2.44±1.09*	= 0.001	15.75

Data are normalized to NG (in %).

* indicates significant differences (p < 0.05) compared to NG.

≈ indicates statistical equivalence for S_NG compared to NG.

P and Χ^2^ values in the right columns display Friedman ANOVA results.

### H-reflex

An increase in loading from UL to OL revealed a significant increase in the H_max_/M_max_-ratio: thereby, a reduction of the loading level below NL led to a decrease of the H_max_/M_max_-ratio and an increased loading level above NL led to an increase of the H_max_/M_max_-ratio (Fig 5). The comparison of S_NL to NL revealed statistical equivalence (Table 3, Fig 5). EMG pre-activation (Fig 5) and M_max_ remained unchanged in response to varied loading levels (Table 3).

## Discussion

The purpose of this study was to elucidate load-dependent adaptations in balance control. The study revealed three main outcomes: (1) Load conditions below and above NL caused an increased COP sway path accompanied by an elevated level of co-contraction reflected by simultaneously activated antagonistic muscle groups encompassing the ankle, knee and hip joint. (2) Modulations in SOL H-reflex sensitivity and joint kinematics occurred opposed for conditions below and above NL: thereby, with an increasing load from UL to OL an increase in H-reflex sensitivity and mean ankle joint excursion as well as a decrease in hip joint excursion were observed. Further, a gradually augmented gravity level from UL to OL caused a gradual shift in the COP frequency distribution from LF to HF. (3) Artificial loading in zero gravity conditions (S_NL) resulted in equivalent neuromuscular and functional values compared to NL. These findings underline that under normal gravity condition balance control is optimally adapted. In contrast, both–UL and OL–led to distinct balance deteriorations and the subjects only achieved postural stability in monopedal stance with great compromises. Although the sample size was small and conclusive statements are based on a careful interpretation of the results, load-dependent changes in compensatory postural regulations and functional consequences can be classified such as follows:

### Adaptations to OL

The SOL H-reflex facilitation observed in our study points towards an increase in spinal excitability [43] and is associated with an exaggerated occurrence of short loop reflexes transmitted via the Ia afferent pathway in the distal muscles of the lower leg [44,31]. In studies investigating the spinal reflex circuitries of the SOL motoneuron pool by means of PNS, it is suggested that an elevated level of spinal excitability leads to a distinctly disturbed control of balance [45,46]. It is further emphasized that this increased excitability of spinal reflexes may cause involuntary ankle joint oscillations [31], concomitant with rapid changes of direction during postural stabilization [38], inducing significantly augmented COP displacements [31]. These findings are in accordance with the observations made in our study, in which proprioceptive sensory input via the Ia afferent reflex circuitry was highly facilitated in OL. Based on recently published work it can be further suggested that the postural oscillations in OL consequently could have contributed to an elevated COP HF band in the TSE [39,38,47,48] and may have resulted in increased mean ankle joint excursions [38]. From a functional perspective, both—augmented ankle joint excursion and COP HF—point towards the predominance of the ankle strategy to organize postural control in OL [47,48]. Vice versa, diminished hip joint excursions indicate that the proximal limb segment is less involved in compensatory balance regulation in OL. In accordance with our study’s findings, numerous studies stated that an increasing postural demand (i.e. reflected by increased COP sways) occurs with a shift from low to high COP frequency components [49–51]. In the light of the latter evidences, our results indicate that subjects use the ankle strategy while stiffening knee and hip joints when challenged by OL to counteract significantly increased sway amplitude.

### Adaptations to UL

Adaptations to UL occurred opposed to those observed in OL. Two aspects may be of considerable importance for the interpretation of the results; the first one deals with balance strategy [52]: As the ankle and knee joint remained rigid whereas hip joint excursions increased, it is suggested that mainly the muscles encompassing the hip joint generated compensatory forces and subjects may have favored the hip strategy using the proximal limb segment to restore equilibrium [53,54,52]. Complementary, considering experiments of Mauritz and Dietz [48] the reduced SOL H-reflex sensitivity and elevated COP LF components observed in our study additionally point towards a reduced ankle strategy: using ankle joint immobilization and ischemic block of short loop reflexes in SOL and GM, Mauritz and Dietz [48] demonstrated that a selective exclusion of the lower leg segment from participating in balance control leads to a shift from ankle to hip strategy concomitant with a predominance of COP LF. Thus, aforementioned findings [53,54,48] are well in line with the results of our study, in which a SOL H-reflex inhibition in UL (without changes in SOL background EMG) was accompanied with an augmented COP LF and increased hip joint excursions indicating the predominance of the hip strategy in UL.

The second aspect deals with a diminished proprioception [40]: in literature it is well documented that the sensory input is of significant relevance for balance control, and that visual, vestibular and proprioceptive afferents are considered to be the major source of information [1,30,2–4]. Loading, however, is known to influence the severity of this kind of information [30,15]. For instance, unloading in a reduced gravity environment—as it occurs during space flight—led to a reduction of proprioceptive information such as those transmitted via muscle spindle, golgi tendon organ and load receptors [30,15]. It is emphasized that sensory information coming from the intact systems of the vestibular organ and vision may compensate for the loss of proprioceptive control [15,55]. Postural control based only on vestibulo-visual feedback, however, is characterized by reduced postural stability accompanied by a peak in COP LF around 1Hz, caused by long neuronal loops transmitted via supraspinal areas such as the cerebellum in balance regulation [48].

From a methodological point of view, one may argue that the choice of test paradigm (single legged balance task opposed to a double support stance), the order of the parabolas or PNS itself could have influenced postural behavior [32,33,31]. Although PNS caused reflective muscle contractions in the Soleus muscle and thus, very small perturbation in anterior direction, COP displacement and LF components showed no difference between the anterior-posterior and medio-lateral direction. As H-reflexes were elicited with 0.25Hz, we conclude that the effect was negligible and gravity-independent (Fig 4). Furthermore, if learning effects interfered in postural control, as the order of the parabolas was not randomized, subjects would have had an advantage in LG after having experienced MG in under loading conditions. Nevertheless, postural sway was largest in LG and thus, largely superimposed by the impact of gravity at least in the challenging postural task of single legged balance (Fig 3).

Taken together, postural stability in OL and UL is achieved contrarily. The ***mechanistic background*** or reasons may be questioned. As the experimental setting provides reliable test conditions between UL, NL and OL [5] without side effects like increased inertia, superficial stabilization [17,6,5] or variable application points of loading [27,28], the most likely candidate is the load-induced torque compensation: a priori, the vertical force acting on the human body in OL is 80% higher than during NL and up to nine times higher than during UL. Consequently, the torque acting on the ankle joint during single limb support is largely elevated [5]. Thus, it can be speculated that rapid and strong muscle forces are required to compensate for the largely augmented joint torques in OL and therefore to restore a given point of equilibrium without falling [56]. This can be achieved by quick reflexive muscle activation elicited in the muscles encompassing the ankle joint close to the support surface. Vice versa, in UL just a fraction of a third (MG) or eighth (LG) of the gravitational force is associated with a substantially reduced ankle joint torque [5]. Hence, to appropriately adjust the COG above the base of support in UL, the proximal segments may control postural equilibrium by slow movements within broad spatial trajectories.

Besides those sharp contrasts, however, commonly for OL and UL an augmented muscle co-contraction reflected by simultaneously activated antagonistic muscle groups encompassing the ankle, knee and hip joint was observed pointing towards a rigid articular stiffening of posture [57–60]. Increased co-contractions may be ascribed to the antigravity function of the leg muscles [56] and have been postulated as a safety strategy to enhance security during single limb support to reduce fall incidences [57–60] while restricting the ability to react precisely to sudden perturbations [61,62]. Thus, it may be speculated that the increased co-contraction is a protective stabilization mechanism serving as a safety strategy in a difficult postural task such as the one-legged stance, however, leading to COP sways equally elevated in OL and UL. Despite commonly increased co-contractions the major findings of the present study are contrary kinematic and neuromuscular characteristics for the test conditions above and below NL. Importantly, when reduced torques in zero gravity conditions are superficially compensated by artificial loading (Fig 2), equivalent neuromuscular and functional characteristics compared to NL can be achieved. Consequently, particularly in view of the latter evidences, it is emphasized that torque compensation is the major underlying parameter for balance control during load variation.

Regarding the ***practical application*** of our findings, the major field of interest besides the space community is the field of training and exercise. In rehabilitation and training, the results of this study could help to establish new therapy-relevant training modalities targeting specific subpopulations within our society. For instance, patients suffering from motor impairments or reduced mobility (elderly, post-surgery, neurological diseases), unable to bear their full bodyweight and incapable of participating in conventional training interventions, could benefit from balance training in under loading. As already described by Freyler et al. [9], balance training with partial loading serves as an appropriate solution improving relevant neuromuscular and functional parameters in an early stage of therapy [9]. Load compensation by means of artificial loading is of particular interest as a countermeasure for astronauts during long term-space flight [15,20]: regarding manned inter-planetary space missions scheduled in the future, impairments in balance control and motor coordination [15,20] in response to changes within the neuromuscular system [63] due to prolonged exposure to zero gravity are the space agencies’ major concerns [19]. In order to maintain health and fitness during space missions, to preserve the capability to execute critical mission tasks and to counteract balance dysfunction, astronauts have to perform physical exercise such as balance training under artificial loading [19]. As neuromuscular and functional values were statistically equivalent in S_NG compared to NG, balance-training regimens performed in artificial loading offer substantial potential to effectively counteract the weightlessness-induced deconditioning of the human body.

A limitation that needs to be considered when interpreting the results of this study is the small sample size of eight subjects. Although subjects were selected carefully and trained before testing to assure repeatability and reproducibility, final conclusive statements require further experiments.

***In conclusion*,** in NL and S_NL postural stability can be easily achieved whereas it is significantly impaired in conditions above and below NL. In view of literature, our results indicate that in OL proprioceptive input transferred via the Ia afferent reflex arc is facilitated and subjects use the ankle strategy while stiffening knee and hip joints to counteract postural imbalance. Concomitantly, high-velocity COP movements refer to rapid changes of direction accompanied by a more tight balance strategy within small spatial trajectories and an elevated postural stiffness. In contrast, postural behavior in UL refers to the hip strategy and an inhibited spinal excitability is attributed to diminished proprioceptive sensory input. COP movements are characterized by slow direction changes within broad spatial trajectories and postural equilibrium may be controlled by the proximal segments to appropriately adjust the COG above the base of support. Besides modulated proprioceptive sensory feedback, altered joint torques due to load variations may have led to the opposed postural strategies. Concerning training and therapy, this aspect seems to be of major relevance: our results could help to establish new therapy and space-relevant training modalities addressing particular strategies and adaptations by means of over loading or under loading conditions [9].
